# Unexpected Reduction of Eu^3+^ Ions to Eu^2+^ on a Nanodiamond Surface: An XPS, NMR, EPR, and Photoluminescence Study

**DOI:** 10.3390/nano16171104

**Published:** 2026-09-01

**Authors:** Natalya Froumin, Alexander Shames, Anastasiya Chizhikova, Maxim Uchaev, Alexander Panich

**Affiliations:** 1 Ilse Katz Institute for Nanoscale Science and Technology, Ben-Gurion University of the Negev, Beer-Sheva 8410501, Israel; 2 Department of Physics, Ben-Gurion University of the Negev, Beer-Sheva 8410501, Israel; 3 Ioffe Institute, St. Petersburg 194021, Russia

**Keywords:** europium, nanodiamond, XPS, NMR, EPR, photoluminescence, reduction of Eu^3+^ ions to Eu^2+^

## Abstract

We present the results of a study of europium-grafted detonation nanodiamond using X-ray photoelectron spectroscopy (XPS), nuclear magnetic resonance (NMR), electron paramagnetic resonance (EPR), and photoluminescence techniques. The material was prepared by mixing an aqueous solution of europium(III) nitrate hexahydrate, Eu(NO_3_)_3_·6H_2_O, with a nanodiamond suspension, resulting in ion exchange between Eu^3+^ ions and protons of surface carboxyl groups and direct grafting of the nanodiamond surface with Eu^3+^ ions. However, after two years of storage under ambient conditions, the compound exhibits the reduction of ~13% of Eu^3+^ ions to the paramagnetic Eu^2+^ state. This unusual finding is discussed in the context of our XPS, EPR, NMR, and photoluminescence data. We propose that this effect can be associated with surface defects and *sp^2^*-hybridized graphene-like fragments that act as electron donors to Eu^3+^ ions. The observed spontaneous partial reduction of Eu^3+^ to Eu^2+^ during long-term storage demonstrates that the oxidation state of surface-grafted europium is not completely stable under ambient conditions. This finding is important because even a partial change in the Eu valence may modify the optical and magnetic properties of Eu-grafted nanodiamonds intended for luminescent, sensing, and quantum applications.

## 1. Introduction

Europium is element #63 on the periodic table, with an atomic mass of 151.96. Naturally occurring europium (^63^Eu) consists of two isotopes, ^151^Eu and ^153^Eu, with natural abundances of 47.8% and 52.2%, respectively [1]. These isotopes show an exceptionally effective neutron absorber used in nuclear reactor control rods due to its high neutron capture cross-section over a wide energy spectrum [2]. Compounds of europium are components of phosphors due to their ability to exhibit luminescence [3,4,5,6,7,8,9,10,11]. They are considered contrast agents for magnetic resonance imaging (MRI) [12,13,14] and materials for bio-imaging applications in medical diagnostics [15]. The radioactive isotopes ^154^Eu and ^155^Eu are used in gamma-ray defect detection [16,17,18].

Europium is not quite an ordinary rare earth element. Most rare earth elements have a highly stable trivalent state in which they lose two 6s electrons and one 4f electron [19,20,21]. One of the few exceptions is europium, which exists in two oxidation states: +3 and +2. Nonmagnetic Eu^3+^ has the electron configuration [Xe] 4f^6^, and paramagnetic Eu^2+^ has the electron configuration [Xe] 4f^7^ [21,22,23,24]. Eu^2+^ has a ground state ^8^S_7/2_, with no orbital angular momentum (L = 0). The magnetic moment of this ion is $\mu \approx 7.94\mu_{B}$. It is found in reducing environments, as well as in compounds with strong donor ligands—chlorides, phosphates, and sulfates (e.g., EuS), and as an impurity ion in various crystals.

The Eu^3+^ ion ([Xe] 4f^6^) is the most common state under oxidizing conditions (i.e., in air) [22,23,24,25]. Complexes with Eu^3+^ are often found in phosphates, oxides, and crystal structures and in minerals and solid compounds naturally found on the Earth’s surface, such as Eu_2_O_3_, EuPO_4_, and Eu^3+^-based dyes and phosphors. They are generally more abundant and stable in nature and most natural environments, whereas Eu^2+^ is found only in highly reducing, specific geochemical conditions (e.g., in early magma or some mineral formations) [23,24,25,26,27,28].

Europium (III) (Eu^3+^) is a typical “hard” Lewis acid, which explains its strong affinity for hard, electronegative, oxygen-containing ligands. In natural systems, it predominantly forms complexes with oxygen donors in oxygen-rich minerals [23,24,25,26]. Eu^3+^ readily forms thermodynamically stable complexes with oxygen-containing ligands such as water, carbonates, and silicates. As a hard acid, Eu^3+^ typically has high coordination numbers, usually eight or nine, allowing it to bind to multiple oxygen atoms simultaneously. Because water is a hard ligand, Eu^3+^ exists predominantly as the hydrated aqua ion in aqueous environments.

Despite this, some studies have observed partial reduction of Eu^3+^ to Eu^2+^ in systems in which electron transfer occurs via intrinsic defects, oxygen vacancies, or electron-rich interfaces. Typical examples, in which the above reduction occurs without an external reducing agent, include oxide phosphors containing oxygen vacancies and rare earth-doped oxides, in which defect-mediated charge compensation occurs [29,30,31,32].

The question is whether a similar phenomenon can be observed in europium-doped carbon compounds. Several studies of Eu^3+^-doped bulk and nanodiamonds [8,9,33], Eu-anchored CNTs [34], and ethylenediaminetetraacetic acid (EDTA) [35] have been reported to date, but they do not demonstrate the characteristic features of Eu^2+^ ions. Sendova et al. [36] studied phosphate glasses melted with a fixed Eu_2_O_3_ content and varying amounts of nanodiamonds and reported carbon-induced reduction of Eu^3+^ to Eu^2+^, concomitant with non-bridging oxygen bond modifications.

In the present study, we investigated detonation nanodiamonds (DNDs) grafted with europium ions directly bound to the diamond surface (thereafter Eu-DNDs). While the as-received Eu-DND sample contained Eu^3+^ ions, our measurements revealed that two years of storage of the sample (thereafter aged Eu-DND) at ambient conditions resulted in partial conversion of the Eu^3+^ ions on the DND surface to the Eu^2+^ state. Since Eu^3+^ complexes are generally more abundant and stable in nature and most environmental environments, this finding is unusual. In this paper, we present our experimental X-ray photoelectron spectroscopy (XPS), electron paramagnetic resonance (EPR), nuclear magnetic resonance (NMR), and photoluminescence (PL) data for these Eu-DND samples and discuss possible mechanisms for the reduction of Eu^3+^ ions to Eu^2+^.

We propose that this effect can be linked to surface defects and *sp^2^*-hybridized graphene-like fragments, which serve as electron donors for Eu^3+^ ions. This carbon-induced reduction of Eu^3+^ to Eu^2+^ is discussed alongside the literature data. The observed reduction of Eu^3+^ to Eu^2+^ during long-term storage demonstrates that the oxidation state of surface-grafted europium is not so stable under ambient conditions. This finding is important as even a partial change in the Eu valence may modify the optical and magnetic properties of Eu-functionalized nanodiamonds intended for luminescent, sensing, and quantum applications.

## 2. Materials and Methods

We investigated a powder sample of europium-grafted nanodiamond, Eu-DND. A non-grafted DND was also studied for comparison. The detonation nanodiamond used in this work was subjected to extensive purification by boiling acid treatment before europium grafting, which substantially removes iron-containing complexes and other para- and ferromagnetic impurities commonly present in as-produced DNDs. EPR and XPS survey spectra do not reveal detectable concentrations of transition-metal contaminants that could account for the observed Eu^3+^ reduction. The samples were prepared as follows: First, a stable suspension of highly purified DND particles was prepared using a previously developed method [37]. The average size of DND particles in the suspension was approximately 4.5 nm, determined using dynamic light scattering (DLS), transmission electron microscopy (TEM), and atomic force microscopy (AFM) [37]. The initial DND suspension was stable, since a high electric potential of −50mV (according to Smoluchowski) was calculated.

Then, a 0.4 wt% Eu-DND suspension was prepared by mixing an aqueous solution of Eu(NO_3_)_3_·6H_2_O with a DND suspension and then removing the unbound Eu^3+^ ions by decantation, similar to other metal-grafted compounds described previously [38,39,40,41]. The resulting mixture was subjected to intense and prolonged ultrasonic irradiation, which promoted disaggregation of DND particles and ion exchange in an aqueous solution between three-charged Eu^3+^ ions and protons of the surface nearest carboxyl groups. As a result, europium ions formed a metal–DND complex with the nanodiamond surface. This is supported by our XPS measurements (see Section 3.1).

To prepare DND and Eu-DND powder, water was removed using a rotary evaporator. According to EDX data (Table 1), the Eu concentration was 0.07 at% (the average value for 6 Eu^3+^ ions grafted onto the DND surface).

Particle size in the suspension was determined using dynamic light scattering (DLS) on a Zetasizer ZS analyzer (Malvern Panalytical, Malvern, UK). Elemental analysis of the powder by energy-dispersive X-ray analysis (EDX) was performed using a Vega 3 SBH scanning electron microscope (Tescan Orsay Holding, Brno, Czech Republic) equipped with an Advanced Aztec Energy EDS analyzer (Oxford Instruments, High Wycombe, UK).

**Figure 1:**
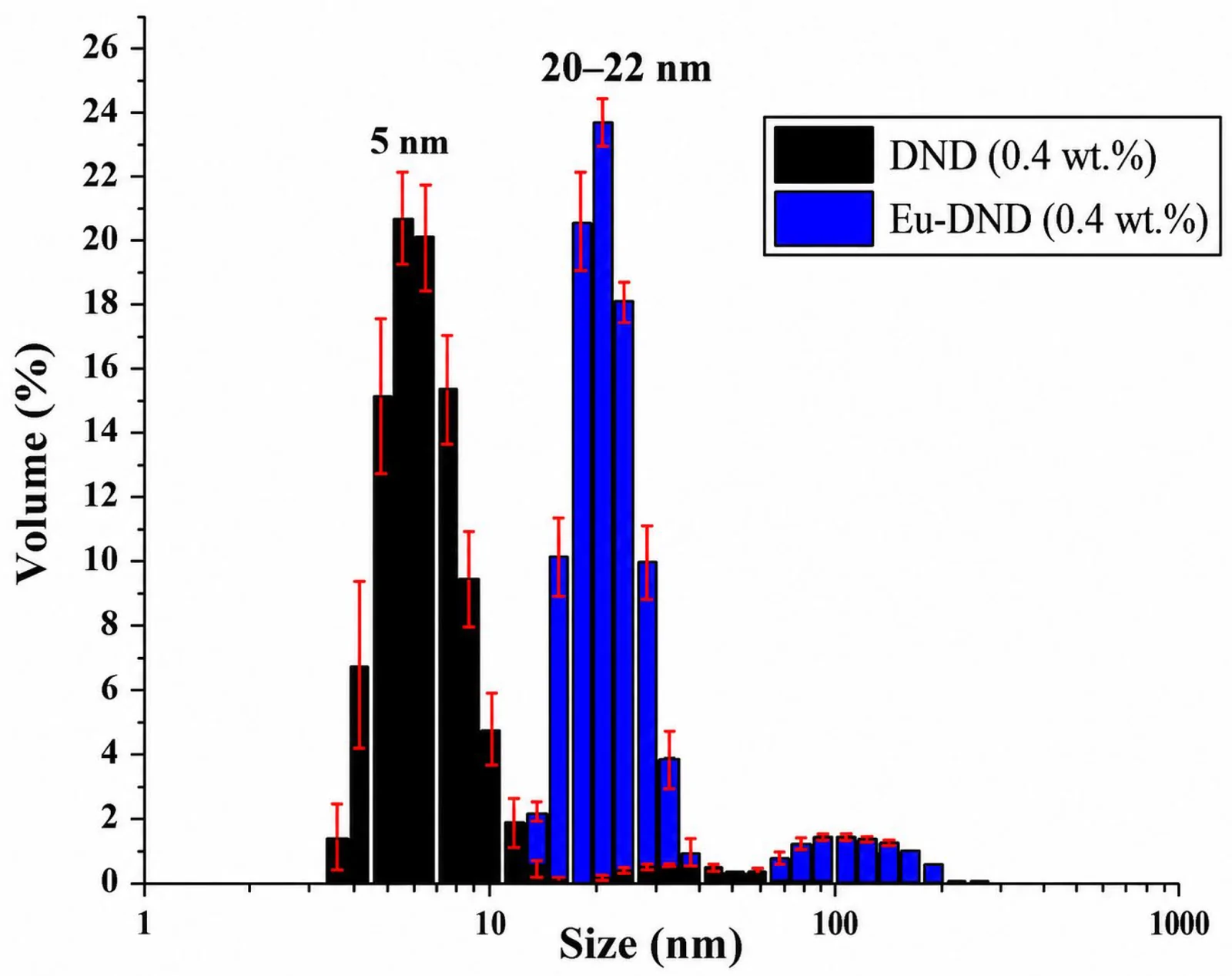
Size distribution of DND and Eu-DND particles. The error bars are shown in red.

The distribution of the particle size of Eu-DND in the suspensions is shown in Figure 1. The diameters of the Eu-DND particles fall in regions of 5, 21, and 100 nm (Figure 1). This indicates aggregation of DND particles during the preparation of Eu-DND samples. The Eu-DND remains stable over extended periods and does not form precipitates.

The Eu-DND sample was stored in a high-purity cylindrical polypropylene tube (TS 9464–015–29508133–2014, Minimed, Suponevo, Russia) with a screw cap. The sample had a volume of 5 mL and was kept in a dry, dark place at room temperature and normal humidity for two years.

XPS measurements were performed using a Thermo Scientific ESCALAB Xi+ ultra-high vacuum (5 × 10^−10^ mbar) X-ray photoelectron spectrometer (East Grinstead, West Sussex, United Kingdom) equipped with a monochromated Al Kα X-ray source (*hν* = 1486.6 eV). The X-ray beam size was 500 μm. Survey spectra were acquired with a pass energy of 150 eV, while high-resolution spectra were recorded with a pass energy of 50 eV and an energy step of 0.1 eV. To correct for charging effects, all binding energies were referenced to the carbon C 1s peak at 284.8 eV. The spectrometer provides an energy resolution of ≤0.45 eV (FWHM of the Ag 3d_5/2_ peak under the manufacturer’s reference conditions, where FWHM is the full width at half maximum). For all spectra, background radiation was corrected using the Smart Background feature of Thermo Fisher Scientific’s Avantage software (version 6.6.0). Atomic ratios were calculated from peak intensity ratios and reported atomic sensitivity factors. The AVANTAGE v6.6.0 Build 00114 Thermo Fisher Scientific software was used for data acquisition and analysis. For band deconvolution and fitting, the corresponding peaks for each element were fitted by using a Lorentzian/Gaussian 30% function mixture and taking FWHM in the range of 1–2.5 eV. The XPS spectra were recorded at different acquisition times to get XPS high-resolution spectra. Electrons from the 3d energy levels were measured for Europium (Eu). All these levels show spin–orbit splitting, which results in two states with different binding energies. Relative intensities were calculated taking into account sensitivity factors for each element.

The ^13^C NMR measurements at room temperature were carried out in static mode on a Bruker Avance III 500 spectrometer (Bruker BioSpin GmbH, Ettlingen, Germany) operating at a resonance frequency of 125.758 MHz with an external magnetic field of 11.7467 T. The spectra were measured using Hahn echo pulse sequence. The spin–lattice relaxation times *T*_1_ were measured using a saturating comb pulse sequence ($\frac{\pi }{2}$ pulse train). Magnetization recoveries were fitted by a stretched exponential function. The duration of the $\frac{\pi }{2}$ pulse was 7.75 μs, with the amplifier power of 60 W. Data processing and simulations were performed using the OriginLab 7.0 software.

Continuous wave (CW) X-band (ν ~ 9.4 and 9.7 GHz) EPR measurements of powder samples were carried out using a Bruker EMX-220 spectrometer (Bruker Biospin GmbH, Rheinstetten, Germany) equipped with Agilent 53150A frequency counter at room temperature (RT, *T*~295 K). Precise determination of *g*-factors (for spin *S* = 1/2 species) and densities of paramagnetic centers *N*_s_ was accomplished by comparison with a reference sample—well-purified detonation nanodiamond powder with *g* = 2.0028(2) and the spins’ content *N*_s_ = 6.3 × 10^19^ spins/g (or 1255 ppm) [42]. Simulations of polycrystalline EPR patterns for Eu^2+^ in various configurations were done using EasySpin package [43].

First XPS, NMR and EPR spectra of as-received Eu-grafted DND samples were measured shortly after the synthesis.

Photoluminescence (PL) spectra were recorded using a confocal optical microscope (NT-MDT, Moscow, Russia) equipped with a 475 nm semiconductor laser with a power of 5 mW. Spectra processing was carried out using IA_P9 software (NT-MDT, Moscow, Russia). The confocal optical system was coupled to a spectrometer SOL Instruments (Minsk, Belarus) and a CCD Andor (Belfast, UK). The emitted light was collected in backscatter geometry using a 100× objective with a numerical aperture (NA) of 0.7 and a diaphragm of 100 μm, ensuring signal collection from a volume of ~5 μm^3^.

Infrared (IR) absorption spectra were obtained using an InfraLUM FT-08 Fourier-transform infrared spectrometer (LUMEX, Saint Petersburg, Russia) equipped with a diffuse reflectance infrared Fourier-transform accessory. The powdered Eu-DND sample was analyzed without the addition of KBr or any pressing. FTIR spectra were collected in the range of 4000 to 400 cm^−1^, with a spectral resolution of 4 cm^−1^, averaging 100 scans for each spectrum.

## 3. Results

### 3.1. XPS Data

We have recently demonstrated the effectiveness of the XPS method for studying nanodiamond particles modified with paramagnetic ions [44]. Now, we apply it to the study of Eu-DND.

**Figure 2:**
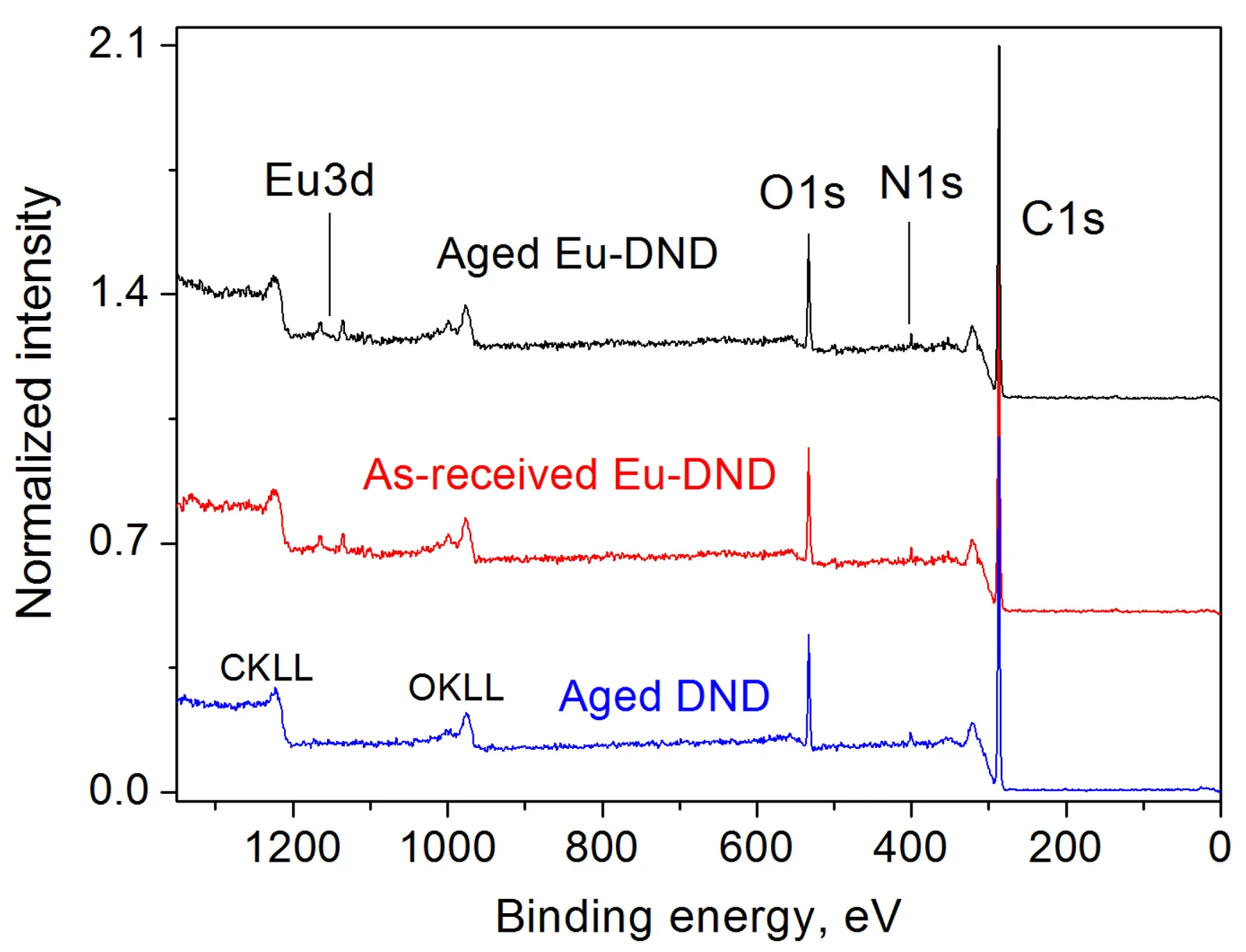
XPS survey spectra of the studied samples. CKLL and OKLL refer to Auger electron transitions for carbon and oxygen.

Surveys of the measured XPS spectra in DND and Eu-DND powder samples are shown in Figure 2.

**Figure 3:**
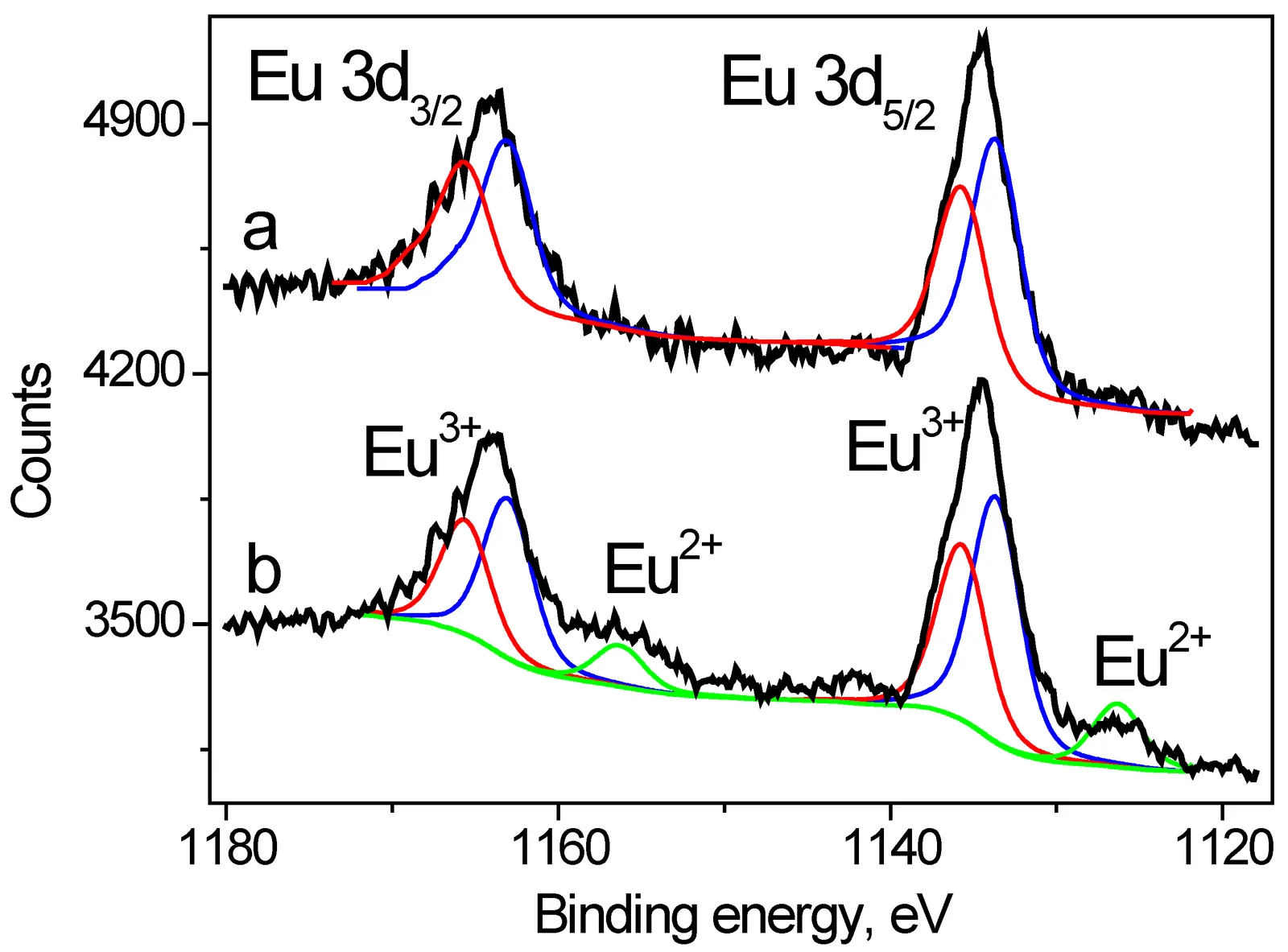
XPS spectra of (**a**) as-received Eu-DND and (**b**) aged Eu-DND samples.

XPS spectra of the Eu3d bands of the as-received and aged Eu-DND samples are shown in Figure 3. They show two intense doublet bands at binding energies of 1163.04, 1165.62 for Eu3d_3/2_ and 1133.64, 1135.71 for Eu3d_5/2_ electron states. The Eu 3d core level spectra are split into a doublet due to spin–orbit coupling with an energy difference of Δ~29.65 eV. According to the literature [29,45,46,47,48,49,50], these bands are attributed to the nonmagnetic Eu^3+^ ion. However, in the aged Eu-DND, two additional weak peaks are observed at 1126.32 and 1156.32 eV, attributed to the paramagnetic Eu^2+^ ion [29,45,46,47,48,49,50]. Table 2 shows the binding energies and atomic percentage of divalent and trivalent europium ions measured by XPS. One can find that approximately 13% of the grafted Eu^3+^ ions were reduced to paramagnetic Eu^2+^ ions over two years of storage. This effect seems to be unusual and requires explanation.

**Figure 4:**
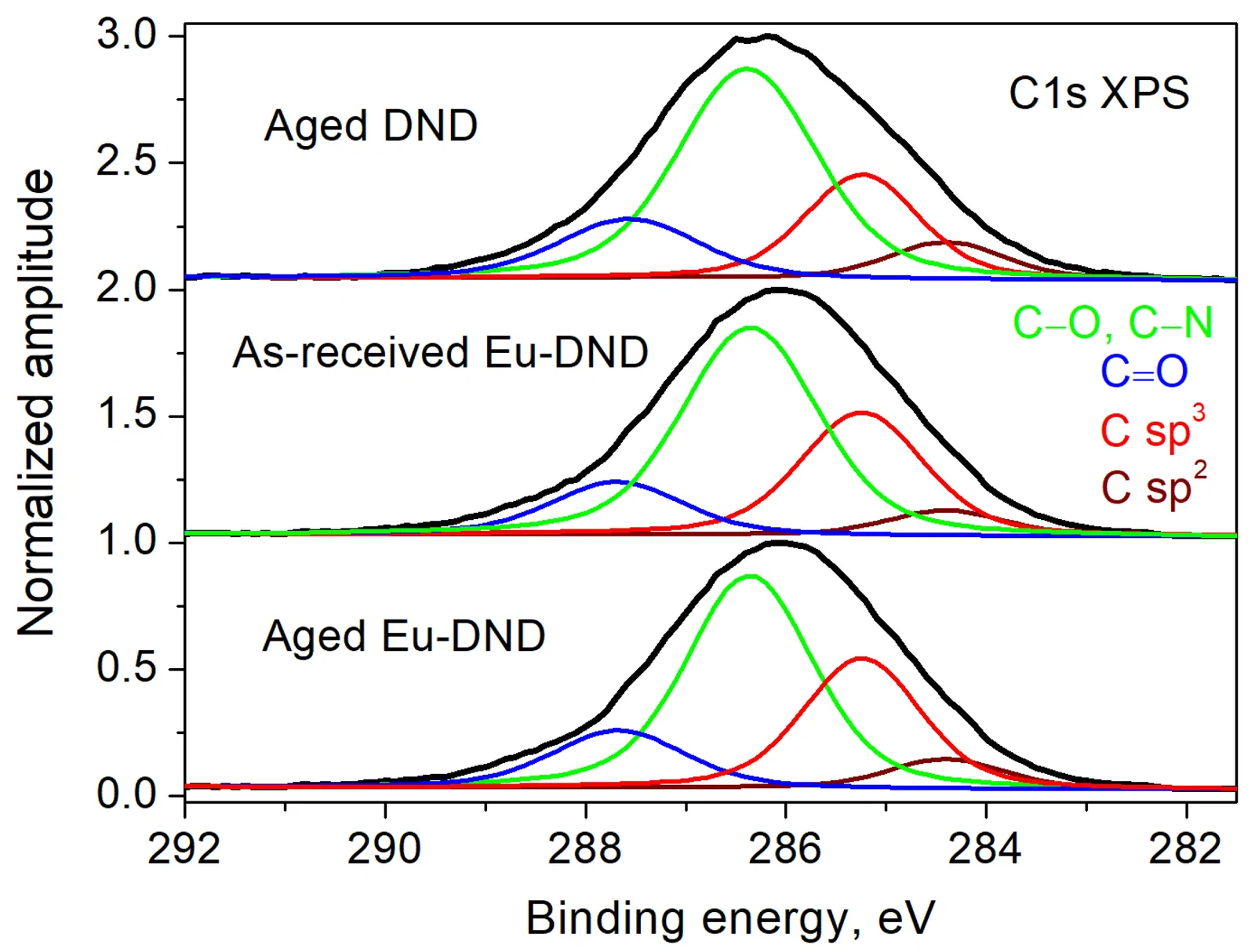
C1s XPS spectra of the aged DND, as-received Eu-DND, and aged Eu-DND samples. C=O, C–O/C–N, C sp^3^, and C sp^2^ bands are shown in blue, green, red, and wine colors, respectively.

**Figure 5:**
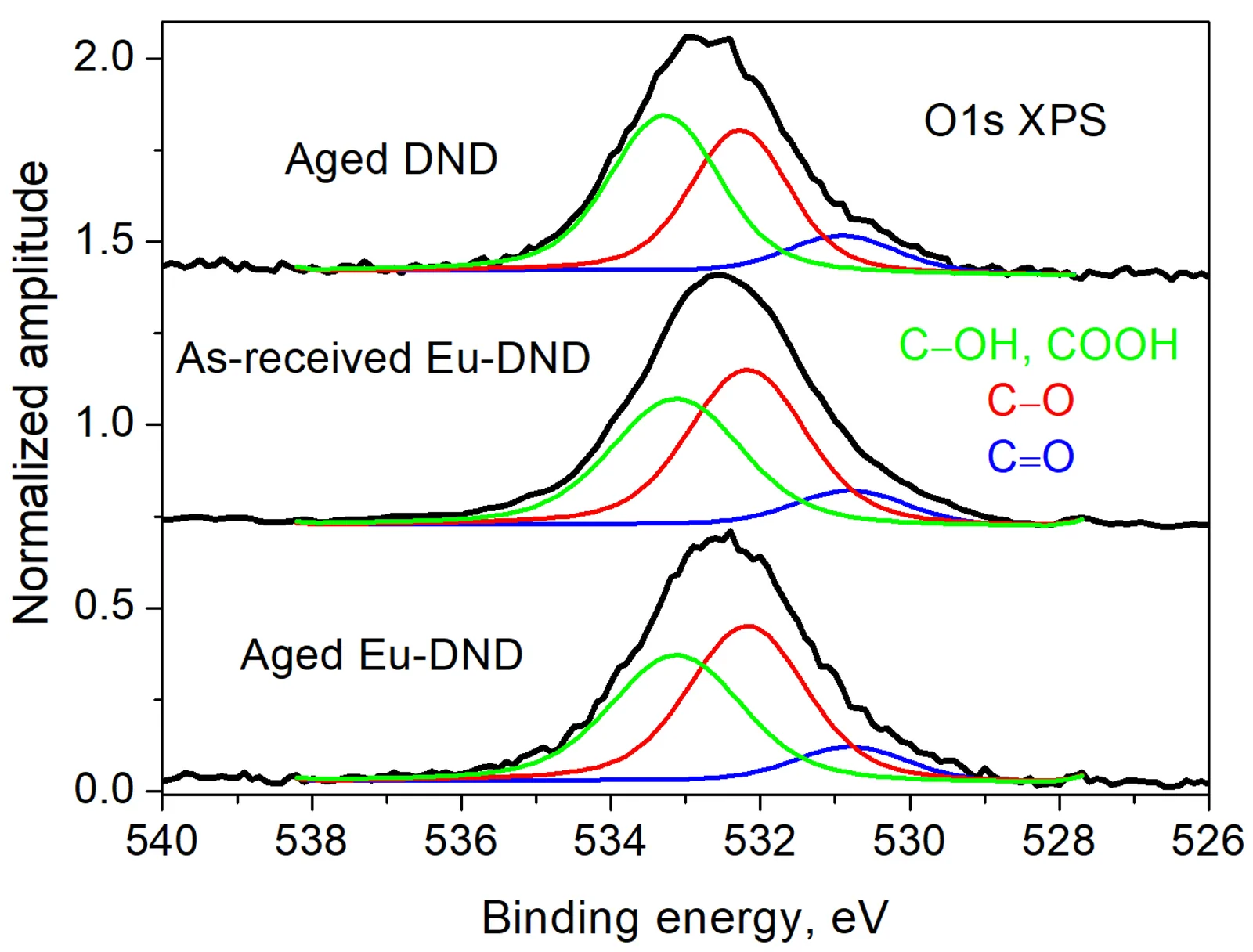
O1s XPS spectra of the aged DND, as-received Eu-DND, and aged Eu-DND samples. C–O, C=O, and C–OH/COOH bands are shown in red, blue and green colors, respectively.

**Figure 6:**
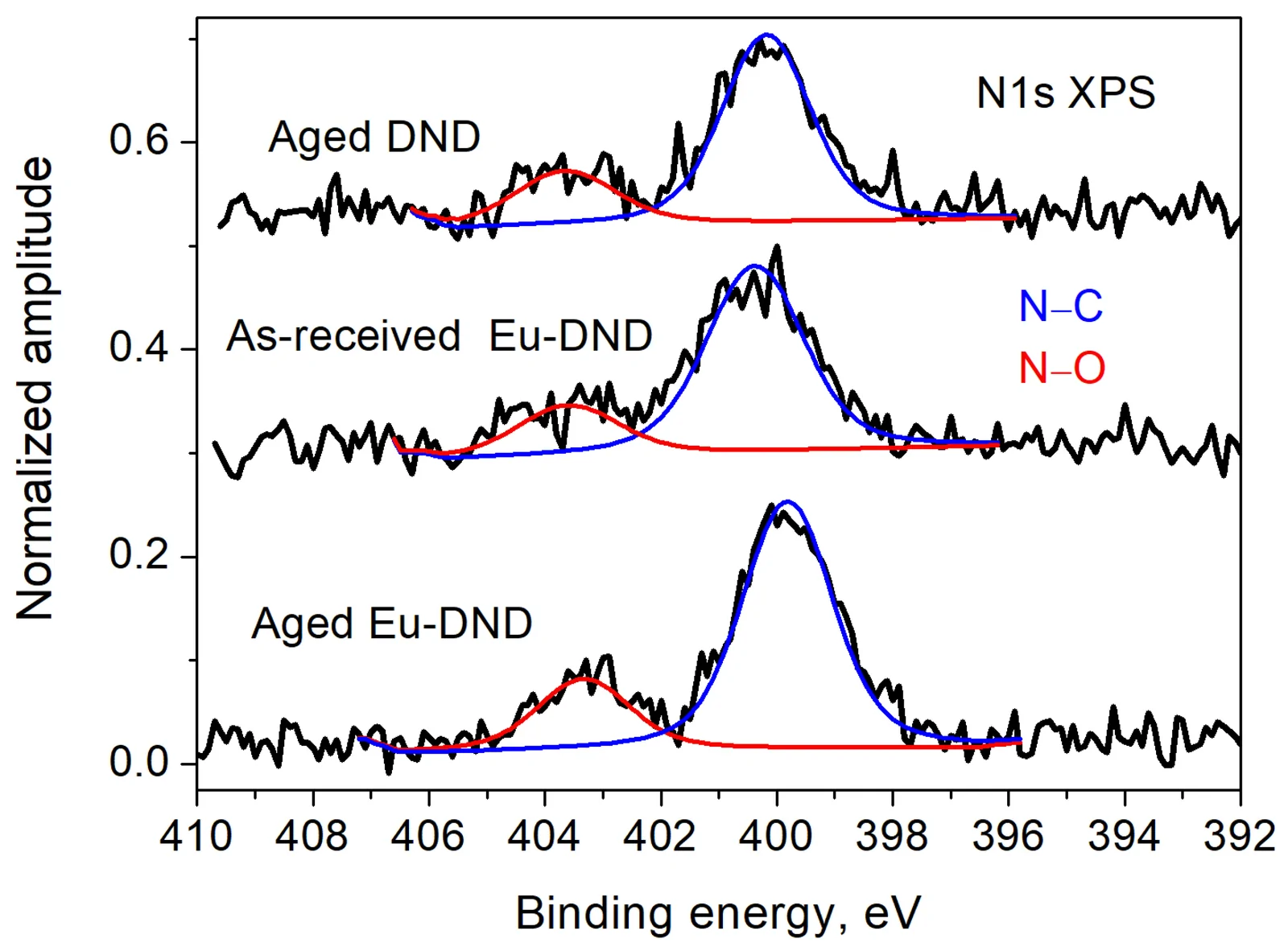
N1s XPS spectra of the aged DND, as-received Eu-DND, and aged Eu-DND samples. N–O and N–C bands are shown in red and blue colors, respectively.

XPS spectra of the C1s, N1s, and O1s bands of the aged DND, as-received and aged Eu-DND samples are shown in Figure 4, Figure 5 and Figure 6. Identification and quantitative analysis of elements in initial DND and Eu-DND are given in Table 3. Detailed quantitative evaluation of C1s, O1s, and N1s peaks in the studied samples and assignment of functional groups according to their binding energies, based on the literature XPS data [44,51,52,53,54,55,56,57,58,59,60,61,62], are provided in Table 4.

The C1s XPS spectra of all studied samples (Figure 4) consist of four bands corresponding to *sp^3^* carbon atoms located in the diamond core, as well as C=O, C–O, CN, and C sp^2^ surface groups. These groups are characteristic of nanodiamonds [44,51,52,53,54,55,56,57,62]. C *sp^2^* atoms are associated with small graphene clusters on the surface, some of which remain after purification.

The O1s XPS spectra of all studied samples (Figure 5) consist of three bands corresponding to the C=O, C–O, and a combination of the C–OH and COOH surface groups. These groups are characteristic of nanodiamonds [44,51,52,53,54,55,56,57]. However, our analysis shows that the incorporation of Eu^3+^ ions leads to a decrease in the intensity of the last peak at ~533.3 eV. This confirms the formation of Eu–O bonds through ion exchange between Eu^3+^ ions and protons of nearby carboxyl groups and indicates that europium is chemically bonded to the surface through oxygen-containing functional groups, rather than simply physically adsorbed. A similar bonding pattern was also observed in nanodiamonds modified with Cu, Co, Gd, and Mn [38,39,40,41,44].

As shown in Figure 6, N1s XPS spectra consist of two components belonging to the N–C and N–O functional groups. This is characteristic of nanodiamonds [44,51,52,54,58,59,60,61]. Since most of the nitrogen atoms in DNDs are located in the diamond core and since they are not connected to europium, no changes are observed in the N1s XPS spectra after grafting Eu ions.

### 3.2. NMR Data

**Figure 7:**
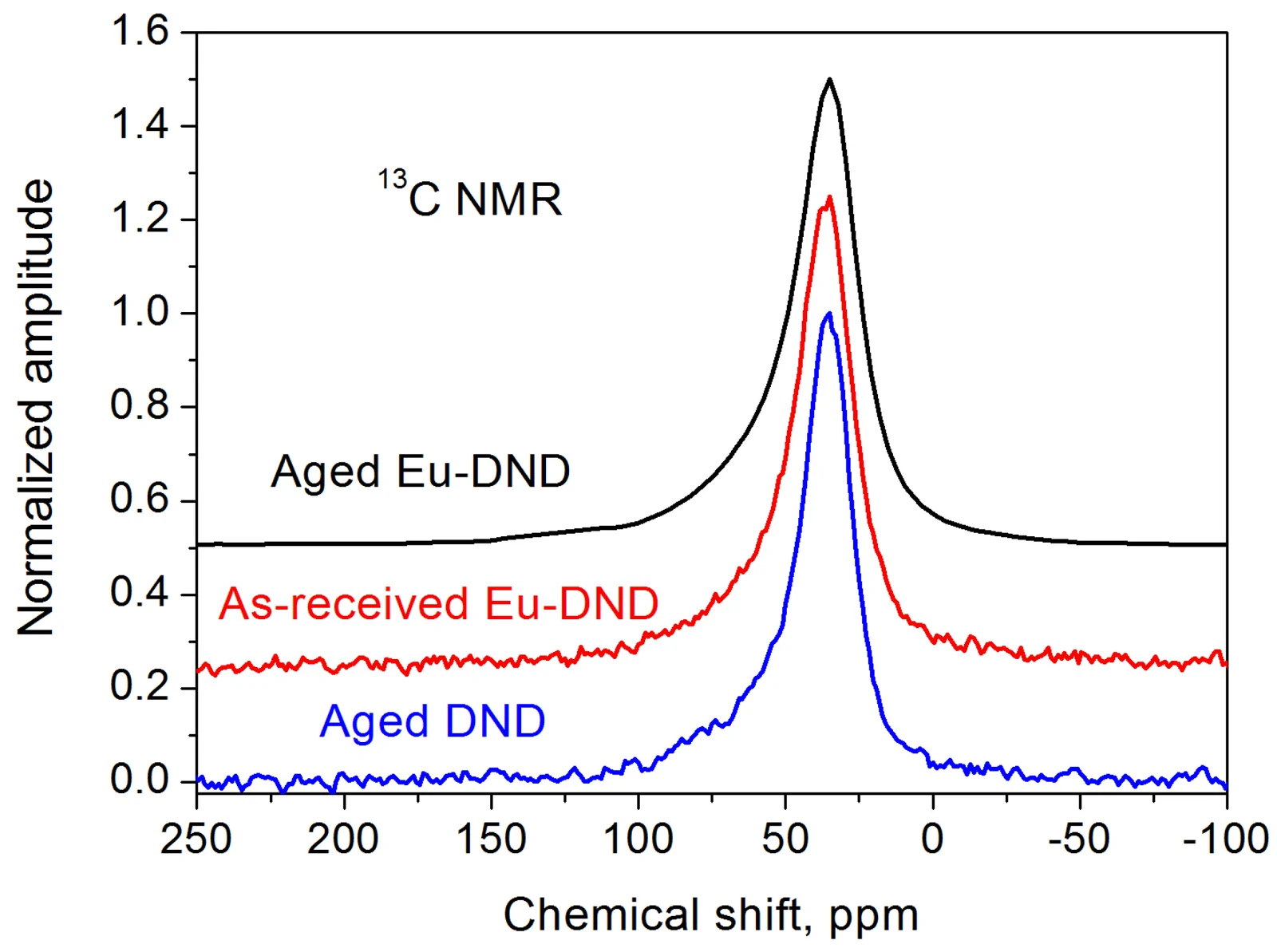
^13^C NMR spectra of the aged DND, as-received Eu-DND, and aged Eu-DND samples.

The ^13^C NMR spectra of the examined samples are shown in Figure 7. As in all DNDs [38,63,64,65,66], the broadening of the spectra primarily results from a combination of core, subsurface, and surface components, along with internal paramagnetic defects. These defects in DNDs mainly include P1 centers and dangling bonds that have electron spins [38,63,64,65,66]. Additionally, a small number of paramagnetic Eu^2+^ ions contribute slightly to the broadening.

At the same time, the interaction of nuclear spins with these localized electron spins creates an effective thermal contact with the lattice modes, forming an effective channel for the nuclear spin–lattice relaxation. This effect significantly accelerates nuclear spin–lattice relaxation. Herewith, according to the theory, the nuclear spin–lattice relaxation time *T*_1_ is inversely proportional to the density of paramagnetic centers [67,68], and the magnetization recovery is described by a stretched exponential function [65,66,69,70,71]. Paramagnetic ions grafted to the nanodiamond surface also accelerate spin–lattice relaxation [38,39,40,41,63,64,65,66,70,71,72]. The appearance of paramagnetic Eu^2+^ ions should also contribute to the relaxation. This is confirmed by our ^13^C NMR measurements, which show a reduction in the relaxation time *T*_1_ in the aged Eu-DND sample compared to the as-received one.

The magnetization recovery in the as-received DND sample was fitted by a stretched exponential function

(1) $$M(t)=M_{\infty }\{1-\exp[-{\left(\frac{t}{T_{1}}\right)}^{\alpha }]\}$$

where $M_{\infty }$ is the equilibrium magnetization, *T*_1_ is the spin–lattice relaxation time, and α is the exponential parameter. The fit yields *T*_1_ = 775 ± 25 ms and α = 0.62. Herewith it can be calculated that the number of the paramagnetic Eu^2+^ ions determined by EPR is smaller than the number of the DND particles, suggesting that DND particles can be either with or without Eu^2+^ ions. Therefore, ^13^C magnetization recovery of the aged Eu-DND sample was fitted by a superposition of two exponential functions:

(2) $$M(t)=M_{1}\{1-\exp[-{\left(\frac{t}{T_{11}}\right)}^{\alpha }]\}+M_{2}\{1-\exp[-{\left(\frac{t}{T_{12}}\right)}^{\alpha }]\}$$

where the first term corresponds to DND particles without Eu^2+^ ions with T_11_ = 775 ± 25 ms, α = 0.62, and the second term corresponds to DND particles with Eu^2+^ ions with T_12_ = 599 ± 21 ms, α = 0.51. The obtained reduction in the spin–lattice relaxation time evidently results from the contribution of the Eu^2+^ions and thus proves the presence of these ions on the DND surface.

### 3.3. EPR Data

**Figure 8:**
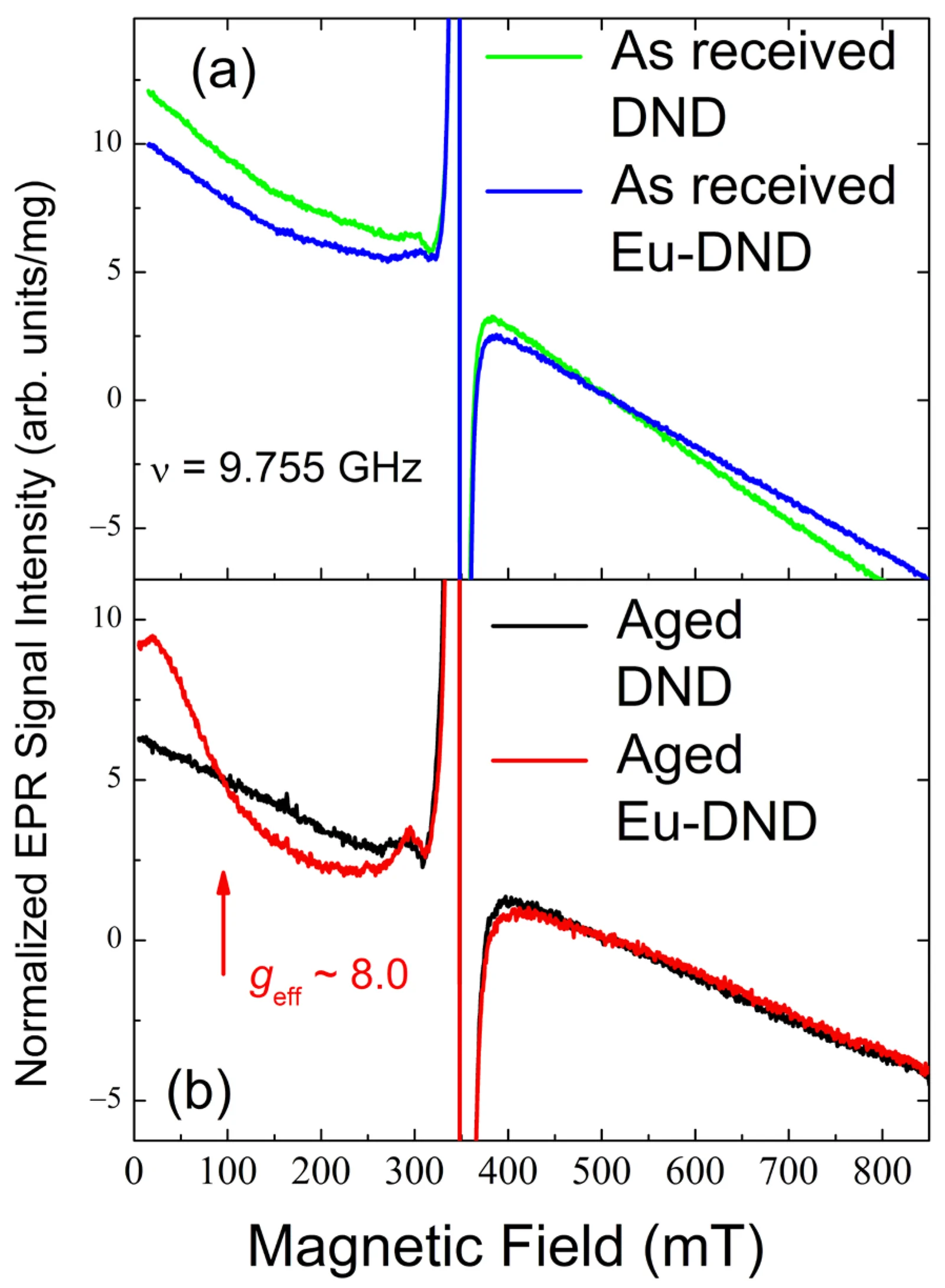
General view RT CW EPR spectra of DND and Eu-DND samples: (**a**) as-received samples, green—pristine DND, blue—Eu-grafted DND; (**b**) aged samples (2 years after), black—DND, red—Eu-DND. Spectra recorded at microwave power *P*_MW_ = 20 mW, 100 KHz magnetic field modulation amplitude A_m_ = 1 mT, receiver gain RG = 10^4^, number of coherent acquisitions n_aq_ = 1, digital resolution N = 2048, microwave frequency ν = 9.755 GHz. No baseline corrections were applied. The arrow indicates the new signal that appeared.

First EPR spectra of as-received DND and Eu-DND samples were measured shortly after the synthesis. General view spectra of both as-received spectra show superposition of a weak broad signal with *g~*2.3 and an intense singlet narrow (line width Δ*H*_pp_ = 0.81(1) mT) Lorentzian-like line with *g* = 2.0028(2)—see Figure 8a. These spectra represent characteristic features of well-purified DND samples [39,40,41,42,63,64,65,66,70,71,72,73]. The intense signals observed may be undoubtedly attributed to structural defects in the interface (shell) layers of DND particle (mostly dangling bonds) exchange coupled with P1 centers in the diamond core. The total content of paramagnetic centers is found to be the same (within the experimental error of 15%) for both samples *N*_s_~6 × 10^19^ spins/g or ~1200 ppm. Thus, no observable difference in spectral EPR features and electron relaxation parameters between as-received pristine and Eu-grafted samples had been observed. Unlike the cases of Cu^2+^-, Mn^2+^-, and Gd^3+^-grafted DNDs [39,40,41,63,72], which clearly revealed EPR signatures of the corresponding paramagnetic ions attached to DND surfaces as well as progressive broadening of the DND-related line with increasing content of grafted ions, the current grafting by nonmagnetic Eu^3+^ ions did not reveal any EPR manifestations.

**Figure 9:**
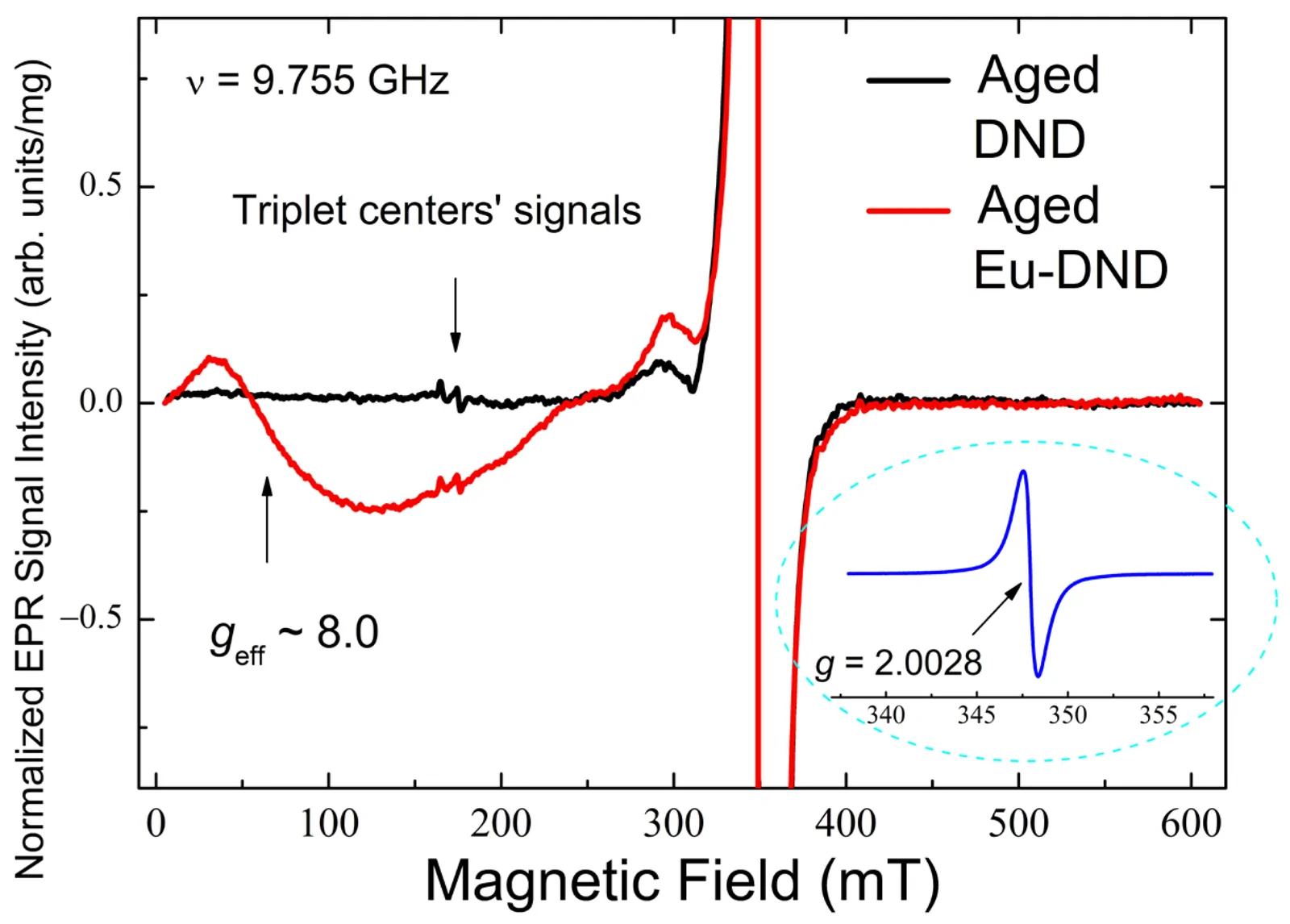
High-resolution RT CW EPR spectra of aged DND (black) and aged Eu-DND (red) samples, *P*_MW_ = 20 mW, A_m_ = 1 mT, receiver gain RG = 10^5^, n_aq_ = 25, N = 2048, ν = 9.755 GHz. Baseline subtracted. The red arrow indicates the new signal at *g*_eff_~8. Black one—“forbidden” signals due to triplet (*S* = 1) NV^−^ centers (*g*_eff_ = 4.25) and multivacancies (*g*_eff_ = 4.00) in DND. The oval-circled inserted shows a zoom of a narrow central line.

However, the EPR measurements of the Eu-DND sample repeated after two years of keeping it at ambient condition (aged Eu-DND) surprisingly revealed the appearance of a new EPR detected feature: a new broad (Δ*H*_p_~200 mT) line in the low-field region centered at *g*_eff_~8. Figure 8b represents the general view EPR spectrum of this sample in comparison with the aged DND sample. The intense narrow lines observed in the aged samples show the same parameters (line width, *g*-factor, paramagnetic centers content) as they were found for as-received samples. Figure 9 shows high-resolution spectra of the low filed region which clearly reveal that the new *g*_eff_~8 signal appeared in the aged Eu-DND sample. Observation of the weak half-field signals, previously attributed to “forbidden” Δ*M*_S_ = 2 transitions in polycrystalline EPR patterns of triplet (*S* = 1) NV^−^ centers *g*_eff_ = 4.25(1) and multivacancies *g*_eff_ = 4.00(1) in DND [73], points out both high sensitivity in detecting the new signal as well as correctness of its *g*-factor estimation. The origin of the *g*_eff_~8 signal in the aged Eu-DND sample cannot yet be unambiguously established by current EPR data. In fact, supposing partial reduction of initially grafted Eu^3+^ ions to Eu^2+^ ions (4*f*^7^, *S* = 7/2, ^151,153Eu^ *I* = 5/2), one could expect observation of Eu^2+^ signals. Indeed, glassy systems containing Eu^2+^ ions in distorted crystal environment may provide low-field signals with *g*_eff_~6 [30,74]. Havlák et al. [75] reported observation of some low-field EPR features mostly within the g~6 region in Eu^2+^-doped Y_3_Al_5_O_12_ (YAG) phosphors. However, EasySpin simulations for Eu^2+^ polycrystalline patterns done with extremely large zero-field splitting parameters |*D*|~0.3–1 cm^−1^ cannot justify the observed EPR signal shift to *g*_eff_~8. On the other hand, some samples in Ref. [75] reveal a broad structureless line in the low-field region with g > 6, which strongly resembles the *g*_eff_~8 signal revealed in the aged Eu-DND sample. These signals have been attributed to some clustered Eu^2+^ entities. This explanation could be considered as a reasonable one for our case, but the observation of these features in [75] occurred at cryogenic temperatures, whereas existence of magnetically ordered Eu^2+^-based entities in the room temperature region seems to be quite puzzling. An alternative explanation of such a signal in aged Eu-DND sample may be the appearance of some impurity iron-based ferromagnetic entities without involving paramagnetic Eu^2+^ ions, having *T*_C_ > RT. However, neither the XPS nor the elemental analysis performed by the EDX/EDS method detected iron in the sample. The latter method allows detection of iron content down to 0.01%.

### 3.4. Photoluminescence Data

**Figure 10:**
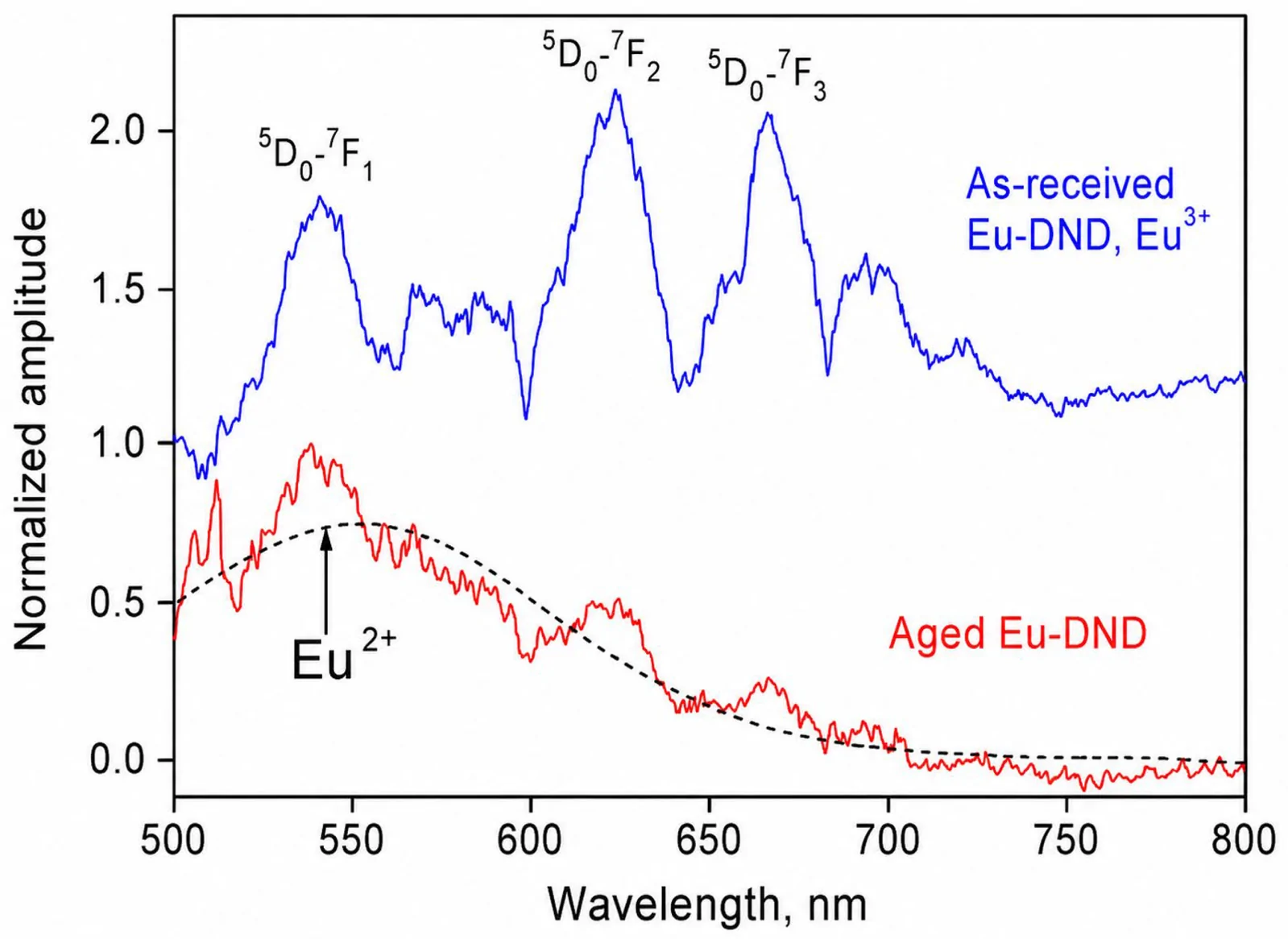
Normalized photoluminescence spectra of as-received Eu-DND (blue) and aged Eu-DND (red) samples measured at an excitation wavelength of 475 nm.

Normalized PL excitation spectra of the as-received and aged Eu-DND samples at the wavelength λ_ex_ = 475 nm after subtracting the DND contribution are shown in Figure 10. The spectrum of the as-received Eu-DND consists of the bands located at 541 nm (^5^D_0_ → ^7^F_1_), 633 nm (^5^D_0_ → ^7^F_2_), and 676 nm (^5^D_0_ → ^7^F_4_) attributed to transitions of Eu^3+^ between excited and ground states [9,33,76]. These bands are also observed in the PL spectrum of the aged Eu-DND sample. In addition, the aged Eu-DND sample exhibits a broad unresolved band from 520 to 700 nm characteristic of the 4f^6^5d^1^ → 4f^7^ transition of the Eu^2+^ ion [29,31,77,78]. This finding confirms the above XPS data that ~13% of the Eu^3+^ ions are reduced to the paramagnetic Eu^2+^ state.

### 3.5. IR Data 

Infrared absorption spectra, their interpretations, and the corresponding references [79,80] are provided in the Supplementary Materials.

## 4. Discussion

Let us consider the physical mechanisms that determine the existence of the 4f^6^ and 4f^7^ electron configurations in Eu ions in various materials, as well as possible mechanisms for the reduction of Eu^3+^ to Eu^2+^ on the DND surface.

According to Hund’s rule, electrons in the sublevels (s, p, d, f) occupy in turn all orbitals with the same spin before pairing occurs. For seven electrons on the f^7^ configuration, each orbital is occupied by one electron with the same spin. This maximizes the exchange energy and minimizes the Coulomb repulsion between electrons.

The Eu^2+^ ion, with the electron configuration [Xe] 4f^7^, has a completely half-filled 4f sublevel (f^7^), which, from a quantum mechanical perspective, provides maximum stability due to exchange energy. The f orbitals have “petal” multi-lobed structures, oriented differently in space, ensuring minimal overlap. In the f^7^ configuration, the electrons are located in different orbitals, maximally separated, and the repulsion between them is minimal. This increases the stability of the f^7^ configuration but does not allow it to withstand external oxidizing agents.

Despite this, Eu^3+^ is the most common complex in nature. The matter is that exchange stabilization is only one component of the overall energy balance. The complete picture also includes thermodynamic stability.

The Eu^3+^ ion has a smaller ionic radius, 95 pm, compared to ~117 pm for Eu^2+^, and a higher charge (+3 versus +2), and it forms stronger ionic bonds with ligands and anions in the lattice compared to Eu^2+^ [81]. Furthermore, the Eu^2+^ ion can be oxidized in the presence of O_2_.

Thus, the exchange stabilization of Eu^2+^ is a real but localized effect at the electron- shell level. Eu^3+^ wins thermodynamically due to the overall energy gain from interactions with the environment, especially in oxygen-containing environments. Eu^3+^ has a 4f^6^ configuration, but the 4f orbitals are shielded, making it highly stable. Next, Eu^3+^ is a “hard” Lewis acid that binds strongly with oxygen-containing ligands, which are dominant in nature.

We note that Eu^2+^ ions were detected in several XPS and photoluminescence studies, often considered as abnormal reduction of Eu^3+^ to Eu^2+^ [29,31,32,48,82,83,84]. Most of the studies were performed with Eu-doped crystals such as Sr_5_(PO_4_)_3_Cl [31], CaAl_2_Si_2_O_8_ [32], Mg_3_Ca_3_(PO_4_)_4_ [84], Ca_10_K(PO_4_)_7_ [29], Sr_5_(PO_4_)_3_F [48], in which trivalent Eu^3+^ ions occupy positions of the divalent Sr^2+^ or Ca^2+^ ions, yielding a charge mismatch between Eu^3+^ and Sr^2+^/Ca^2+^ ions, which initiates the Eu^3+^ → Eu^2+^ transformation. This requires electron transfers from chemical elements of the host lattice towards the rare earth element.

In our study, we have found that europium ions grafted on the surface of detonation nanodiamonds, initially in the 3+ valence state, are partially (around 13%) transformed into 2+ ions during long-term storage under normal conditions. This is a very unusual process. Let us discuss the chemical processes that could lead to this transformation.

First, DND is not an inert substrate. Its surface contains carboxyl (–COOH), hydroxyl (–OH), carbonyl, lactone, anhydride, and epoxy groups, graphene-like domains (*sp^2^* carbon), as well as radical centers and defects. These defect states can act as long-lived electron reservoirs. Over long timescales, electrons can tunnel or hop to coordinated Eu^3+^ ions: Eu^3+^ + e^−^ → Eu^2+^. The surface chemistry of DND makes the aforementioned process possible.

A possible reduction mechanism for DND particles involves charge transfer from *sp^2^* graphene-like surface domains. The presence of these domains has been confirmed by our XPS measurements, as shown in Figure 4 and Table 4. Since Eu^3+^ is directly grafted to the surface and coordinated near these domains, the graphene-like *sp^2^* regions on the DND surface can function as electron donors:

$$Eu^{3+}+[C_{sp^{2}}]\to Eu^{2+}+{\left[C_{sp^{2}}\right]}^{+}$$

This process is thermodynamically possible if the ionization potential of the graphene domain is lower than the redox potential of the Eu^3+^/Eu^2+^ pair (−0.36 V). Aromatic/graphite systems are reducing agents known for rare earth elements in organic chemistry (Birch reactions and analogs). This is a slow process, as charge transfer through a solid is a process with an activation barrier, depending on the geometry of the Eu^3+^ contact with the *sp^2^* domain. Such carbon-induced Eu^3+^ → Eu^2+^ reduction was observed in ref. [36], which discusses nanodiamond carbon as a chemical reductant for Eu^3+^ ions.

The effect can also be realized due to the carbon radicals of DND, especially after detonation synthesis. DND is known to contain two main types of paramagnetic centers: subsurface radicals with a g-factor of ~2.0028, which are *sp^3^* carbons with a dangling bond, and graphene domains with a g-factor of ~2.0025, which are delocalized π-radicals.

During prolonged storage, radicals initially stored in the subsurface region can migrate to the surface Eu^3+^ ions through defects in the crystal lattice and act as reducing agents, resulting in a slow time-delayed process. Such processes can also be stimulated in DND agglomerates, where the particles are in contact with each other. And the DND surface creates a local reducing microenvironment, making the Eu^2+^ ion stable in a crystal lattice. It is well known as the europium anomaly.

Intense XPS Eu^2+^ bands at 1125.6 and 1154.75 eV are observed in europium sulfide EuS, in which divalent sulfur S^2−^ ion accepts two electrons from the divalent Eu^2+^ [50]. In compounds with no divalent anions, only partial reduction was obtained. For example, Sipina et al. [29] observed and discussed in detail an abnormal reduction of Eu^3+^ → Eu^2+^ in air in Ca_9-x_Mn_x_Eu(PO_4_)_7_ using photoluminescence (PL) and XPS. Eu^3+^ shows partial reduction and coexistence of Eu^2+^ and Eu^3+^ states. The Eu3d spectra of the samples show a doublet of lines Eu3d_5/2_ and Eu3d_3/2_ at 1135.0 and 1164.5 eV binding energies. These values are typical for the Eu^3+^ ion [45,46,50]. The additional Eu3d_5/2_ component with binding energy 1124.4 eV was attributed to Eu^2+^ [29,45,47,50]. The quantity of divalent europium in Ca_8_MnEu(PO_4_)_7_ was about 5% of the total europium content.

Eu^3+^ → Eu^2+^ reduction in inorganic phosphors was observed in several studies [30,31,32,82,83,84], leading to the coexistence of two types of europium oxidation states. There is no information on the full reduction of Eu^3+^ to Eu^2+^ in non-reduction media.

We note that since our sample was stored in a dark place, a photoreduction mechanism, when light excites an electron from a ligand orbital to a metal atom, is not relevant in the case in question.

The fact that we observe a stable mixed-valence state of Eu ions means that the DND surface creates a coordination environment that thermodynamically stabilizes Eu^2+^—likely through the specific geometry and donor properties of the subsurface radical centers and defects.

We note that although some trace metallic impurities below the XPS and EPR detection limit cannot be completely excluded, their contribution is expected to be negligible because the nanodiamond was extensively acid-purified and the reduction develops only after prolonged storage rather than immediately after synthesis. If residual metallic impurities were the dominant electron source, one would expect reduction to occur during or shortly after grafting rather than only after prolonged aging. Instead, our results are more consistent with a slow electron transfer process involving intrinsic defect states of nanodiamond, particularly subsurface dangling bond-related electronic states previously identified by EPR, together with gradual structural relaxation of the surface coordination environment. Thus, the available experimental evidence does not support impurities as the origin of the observed Eu^3+^ → Eu^2+^ conversion. 

## 5. Conclusions

We investigated detonation nanodiamonds with Eu^3+^ ions directly grafted onto their surface using X-ray photoelectron spectroscopy (XPS), nuclear magnetic resonance (NMR), electron paramagnetic resonance (EPR), and photoluminescence (PL) spectroscopy. The as-prepared Eu-DND sample contained exclusively Eu^3+^ ions. Unexpectedly, after two years of storage under ambient conditions, approximately 13% of the Eu^3+^ ions underwent spontaneous reduction to the paramagnetic Eu^2+^ state. This previously unreported phenomenon is supported by complementary XPS, EPR, NMR, and PL measurements. Based on the experimental results, we propose that the reduction may be facilitated by surface defects and *sp^2^*-hybridized carbon atoms acting as electron donors.

The principal outcome of this work is the experimental demonstration of spontaneous partial Eu^3+^ → Eu^2+^ reduction in europium-functionalized detonation nanodiamonds during long-term storage. Although the detailed reduction mechanism and its kinetics remain to be established, the present findings provide a foundation for future investigations aimed at elucidating the underlying electron transfer processes and the factors governing the stability of europium oxidation states on nanodiamond surfaces.

## Figures and Tables

**Table 1:** Elemental content * of Eu-DND sample in weight and atomic percentages according to the EDS data.

Eu-DND	C	O	Eu	Cl	Ti
Wt%	90.24	8.65	0.92	0.14	0.05
At%	93.15	6.72	0.07	0.05	0.01

* The EDX method has limitations in determining light elements such as nitrogen, as the nitrogen peak often overlaps with the carbon peak, making quantification of nitrogen by this technique difficult.

**Table 2:** Atomic percentage of divalent and trivalent europium ions measured by XPS.

Sample	Ions	Binding Energies of Eu3d_5/2_ Peaks	Atomic %
As-received Eu-DND	Eu^3+^	1133.69	58.05
	Eu^3+^	1135.75	41.95
Aged Eu-DND	Eu^3+^	1133.64	50.77
	Eu^3+^	1135.71	36.27
	Eu^2+^	1126.32	12.96

**Table 3:** Identification and quantification of elements in initial DND and Eu-DNDs.

Sample	Level	Peak BE, eV	Atomic %	SF
Aged DND	C1s	286.71	85.31	1.000
	N1s	400.51	1.90	1.676
	O1s	532.74	12.79	2.881
As-received Eu-DND	C1s	286.15	87.48	1
	N1s	399.80	2.04	1.676
	O1s	532.27	10.23	2.881
	Eu3d_5/2_	1134.50	0.25	48.796
Aged Eu-DND	C1s	286.07	87.44	1
	N1s	399.87	2.07	1.676
	O1s	532.24	10.25	2.881
	Eu3d_5/2_	1134.49	0.25	48.796

**Table 4:** Detailed quantification of C1s, O1s, and N1s bands in DND and Eu-DND samples. The assignment of functional groups according to their binding energies follows those accepted in the literature [44,45,46,47,48,49,50,51,52,53,54,55,56,57,58,59,60,61,62].

*Sample*	*C1s Peaks*	*C1s, at%*	*Group*
Aged DND	284.4	8.15	C *sp*^2^
	285.24	22.27	C–C *sp^3^*
	286.39	54.41	C–O, C–N
	287.57	15.17	C=O
As-received Eu-DND	284.4	7.35	C *sp^2^*
	285.54	26.90	C-C *sp^3^*
	286.56	52.58	C–O, C–N
	287.8	13.17	C=O
Aged Eu-DND	284.4	6.57	C *sp^2^*
	285.24	28.74	C-C *sp^3^*
	286.36	50.72	C–O, C–N
	287.67	13.97	C=O
	***O1s peaks***	***O1s, at%***	***Group***
Aged DND	530.91	12.19	C=O
	532.27	39.58	C-O
	533.28	48.23	C–OH, COOH
As-received Eu-DND	530.98	10.52	C=O
	532.28	47.01	C-O
	533.28	42.47	C–OH, COOH
Aged Eu-DND	530.8	10.54	C=O
	532.17	46.97	C–O
	533.12	42.49	C-OH, COOH
	***N1s peaks***	***N1s, at%***	***Group***
Aged DND	400.49	74.89	N–C
	403.65	25.11	N–O
As-received Eu-DND	400.01	77.65	N–C
	403.42	22.35	N–O
Aged Eu-DND	399.82	77.17	N–C
	403.34	22.83	N–O

## Data Availability

The original contributions presented in this study are included in the article/Supplementary Material. Further inquiries can be directed to the corresponding author.

## Supplementary Materials

The following supporting information can be downloaded at: https://www.mdpi.com/article/10.3390/nano16171104/s1. The Supplementary Materials (Figure S1. IR spectra of DND and Eu-DND powders) are given in a separate file.

## References

[1] Casali N., Nagorny S.S., Orio F., Pattavina L., Beeman J.W., Bellini F., Cardani L., Dafinei I., Domizio S.D., Vacri M.L.D. (2014). Discovery of the ^151^Eu α Decay. J. Phys. G Nucl. Part. Phys..

[2] Leinweber G., Barry D.P., Burke J.A., Rapp M.J., Block R.C., Danon Y., Geuther J.A., Saglime F.J. (2014). Europium Resonance Parameters from Neutron Capture and Transmission Measurements in the Energy Range 0.01–200eV. Ann. Nucl. Energy.

[3] Bünzli J.-C. (2010). Europium in the Limelight. Nat. Chem..

[4] Ruegenberg F., García-Fuente A., Seibald M., Baumann D., Peschke S., Urland W., Meijerink A., Huppertz H., Suta M. (2021). Chasing Down the Eu^2+^ Ions: The Delicate Structure−Property Relationships in the Ultra-Narrow Band Phosphor K_1.6_Na_2.1_Li_0.3_[Li_3_SiO_4_]_4_:Eu^2+^. Adv. Opt. Mater..

[5] Xie Q., Li B., He X., Zhang M., Chen Y., Zeng Q. (2017). Correlation of Structure, Tunable Colors, and Lifetimes of (Sr, Ca, Ba)Al_2_O_4_:Eu^2+^, Dy^3+^ Phosphors. Materials.

[6] Dotsenko V.P., Levshov S.M., Berezovskaya I.V., Stryganyuk G.B., Voloshinovskii A.S., Efryushina N.P. (2011). Luminescent Properties of Eu^2+^ and Ce^3+^ Ions in Strontium Litho-Silicate Li_2_SrSiO_4_. J. Lumin..

[7] Marinho J.P.N., Serrano M.K.S.R.C., Amaral K.P., Oliveira A.F., Vieira L.A.F., Apostolos R.C.R., Sousa E.M.B. (2025). Hydroxyapatite Nanomaterials Co-Doped with Gd^3+^ and Eu^3+^ for Luminescent Imaging and Targeted Drug Delivery. Acad. Mater. Sci..

[8] Lebedev V.T., Shakhov F.M., Vul A.Y., Zakharov A.A., Zinoviev V.G., Orlova V.A., Fomin E.V. (2023). X-Ray Excited Optical Luminescence of Eu in Diamond Crystals Synthesized at High Pressure High Temperature. Materials.

[9] Yudina E.B., Aleksenskii A.E., Bogdanov S.A., Bukalov S.S., Leites L.A., Radishev D.B., Vikharev A.L., Vul’ A.Y. (2022). CVD Nanocrystalline Diamond Film Doped with Eu. Materials.

[10] Tsukerblat B., Nazarov M., Palii A. (2026). Probing Multiphonon Luminescence Bands in Eu^2+^-Doped Thiogallate Phosphors via Spectral Moments Approach. J. Appl. Phys..

[11] Werts M.H.V. (2005). Making Sense of Lanthanide Luminescence. Sci. Prog..

[12] Garcia J., Neelavalli J., Haacke E.M., Allen M.J. (2011). EuII-Containing Cryptates as Contrast Agents for Ultra-High Field Strength Magnetic Resonance Imaging. Chem. Commun..

[13] Garcia J., Kuda-Wedagedara A.N.W., Allen M.J. (2012). Physical Properties of Eu^2+^ -Containing Cryptates as Contrast Agents for Ultrahigh-Field Magnetic Resonance Imaging. Eur. J. Inorg. Chem..

[14] Heffern M.C., Matosziuk L.M., Meade T.J. (2014). Lanthanide Probes for Bioresponsive Imaging. Chem. Rev..

[15] Woo H.-J., Lee M., Kumar P.P.P., Lim J.-M., Kim T. (2025). Dendritic Mesoporous Silica Structuring of Eu^3+^ -Doped Ca_2_SiO_4_ Nanophosphors for Bioimaging and Luminescent Applications. ACS Appl. Nano Mater..

[16] Kulp W.D. (2005). An Investigation of ^154^Eu as a High-Precision Multi-γ-Ray Intensity Calibration Standard for Detector Arrays. Proceedings of the AIP Conference Proceedings.

[17] Mahabadi M., Ranjbar H., Islami-Rad S.Z., Boustani E. (2026). Feasibility of Simultaneous ^152^Eu and ^154^Eu Radioisotope Production from Natural Europium for Gamma Spectrometry Calibration. Nucl. Anal..

[18] Mahabadi M., Ranjbar H., Islami-Rad S.Z., Bagheri R. (2025). Optimized Production of Dual-Isotope Sources for Gamma Detector Calibration. Sci. Rep..

[19] Johnson D.A., Nelson P.G. (2018). Valencies of the Lanthanides. Found. Chem..

[20] Bünzli J.-C.G. (2014). Review: Lanthanide Coordination Chemistry: From Old Concepts to Coordination Polymers. J. Coord. Chem..

[21] Jaffe P.M., Banks E. (1955). Oxidation States of Europium in the Alkaline Earth Oxide and Sulfide Phosphors. J. Electrochem. Soc..

[22] Lewandowski E.C., Arban C.B., Deal M.P., Batchev A.L., Allen M.J. (2024). Europium(ii/iii) Coordination Chemistry toward Applications. Chem. Commun..

[23] Dorenbos P. (2005). Valence Stability of Lanthanide Ions in Inorganic Compounds. Chem. Mater..

[24] Tricoire M., Mahieu N., Simler T., Nocton G. (2021). Intermediate Valence States in Lanthanide Compounds. Chem. A Eur. J..

[25] Dereń P.J., Stefańska D., Ptak M., Wiśniewski P. (2021). Method to Measure the Degree of Reduction of Eu^3+^ to Eu^2+^: How Anion and Cation Vacancies Influence the Degree of Reduction. J. Phys. Chem. C.

[26] Issabekova S., Belgibayeva D., Amerkhanova S., Satayeva Z., Abilova G., Almuratova K., Aikenova N., Sharipova L. (2026). Thermodynamic Parameters and Coordination Behavior of Eu(III) Complexes with Tartrate and Oxalate Ligands: Study Using NMR and Potentiometry Methods. Inorganics.

[27] Vicentini G., Zinner L.B., Zukerman-Schpector J., Zinner K. (2000). Luminescence and Structure of Europium Compounds. Coord. Chem. Rev..

[28] Bünzli J.-C.G., André N., Elhabiri M., Muller G., Piguet C. (2000). Trivalent Lanthanide Ions: Versatile Coordination Centers with Unique Spectroscopic and Magnetic Properties. J. Alloys Compd..

[29] Sipina E.V., Spassky D.A., Krutyak N.R., Morozov V.A., Zhukovskaya E.S., Belik A.A., Manylov M.S., Lazoryak B.I., Deyneko D.V. (2023). Abnormal Eu^3+^ → Eu^2+^ Reduction in Ca_9−x_Mn_x_Eu(PO_4_)_7_ Phosphors: Structure and Luminescent Properties. Materials.

[30] Malchukova E., Boizot B. (2010). Reduction of Eu^3+^ to Eu^2+^ in Aluminoborosilicate Glasses under Ionizing Radiation. Mater. Res. Bull..

[31] Chen J., Liang Y., Zhu Y., Liu S., Li H., Lei W. (2019). Abnormal Reduction of Eu^3+^ to Eu^2+^ in Sr_5_(PO_4_)_3_Cl:Eu Phosphor and Its Enhanced Red Emission by the Charge Compensation. J. Lumin..

[32] Dai W.B. (2014). Mechanism of the Reduction and Energy Transfer between Eu^2+^ and Eu^3+^ in Eu-Doped CaAl_2_Si_2_O_8_ Materials Prepared in Air. J. Mater. Chem. C.

[33] Magyar A., Hu W., Shanley T., Flatté M.E., Hu E., Aharonovich I. (2014). Synthesis of Luminescent Europium Defects in Diamond. Nat. Commun..

[34] Qi X., Zhang W., Yang N., Yang D., Bychko I., Chen M., Wang Y., Han D., Huang L., Jiang H. (2021). Direct Anchoring of Eu^3+^ Complex to Derivative Surfaces of Multi-Wall Carbon Nanotubes (Eu@DSCNTs) for Linear Fluorescence Nanomaterials. J. Alloys Compd..

[35] Ji T., Fan P., Li X., Mei Z., Mao Y., Tian Y. (2019). EDTA-Bonded Multi-Connected Carbon-Dots and Their Eu^3+^ Complex: Preparation and Optical Properties. RSC Adv..

[36] Sendova M., Jiménez J.A. (2022). Nanodiamond-Induced Modifications of Eu-Doped Phosphate Glasses toward Photonic Applications: A Synergistic Physico-Chemical Approach. Mater. Adv..

[37] Aleksenskiy A.E., Eydelman E.D., Vul’ A.Y. (2011). Deagglomeration of Detonation Nanodiamonds. Nanosci. Nanotechnol. Lett..

[38] Panich A.M. (2017). Nuclear Magnetic Resonance Studies of Nanodiamond Surface Modification. Diam. Relat. Mater..

[39] Panich A.M., Shames A.I., Medvedev O., Osipov V.Y., Aleksenskiy A.E., Vul’ A.Y. (2009). Magnetic Resonance Study of Detonation Nanodiamonds with Surface Chemically Modified by Transition Metal Ions. Appl. Magn. Reson..

[40] Panich A.M., Shames A.I., Sergeev N.A., Osipov V.Y., Alexenskiy A.E., Vul’ A.Y. (2016). Magnetic Resonance Study of Gadolinium-Grafted Nanodiamonds. J. Phys. Chem. C.

[41] Panich A.M., Shames A.I., Aleksenskii A.E., Yudina E.B., Vul’ A.Y. (2021). Manganese-Grafted Detonation Nanodiamond, a Novel Potential MRI Contrast Agent. Diam. Relat. Mater..

[42] Osipov V.Y., Shames A.I., Enoki T., Takai K., Baidakova M.V., Vul’ A.Y. (2007). Paramagnetic Defects and Exchange Coupled Spins in Pristine Ultrananocrystalline Diamonds. Diam. Relat. Mater..

[43] Stoll S., Schweiger A. (2006). EasySpin, a Comprehensive Software Package for Spectral Simulation and Analysis in EPR. J. Magn. Reson..

[44] Panich A., Froumin N., Aleksenskii A., Chizhikova A. (2025). XPS Study of Grafting Paramagnetic Ions onto the Surface of Detonation Nanodiamonds. Nanomaterials.

[45] xpsdatabase.net. https://xpsdatabase.net/europium-eu-z63-europium-compounds/?v=4605f628f91d.

[46] Bruncková H., Kaňuchová M., Kolev H., Múdra E., Kovalčiková A., Medvecký Ľ. (2020). X-Ray Photoelectron Spectroscopy Study of Europium Niobate Thin Film Prepared by Chemical Solution Deposition. Powder Metall. Prog..

[47] Mariscal A., Quesada A., Tarazaga Martín-Luengo A., García M.A., Bonanni A., Fernández J.F., Serna R. (2018). Europium Monoxide Nanocrystalline Thin Films with High Near-Infrared Transparency. Appl. Surf. Sci..

[48] Swart H.C., Nagpure I.M., Ntwaeaborwa O.M., Fisher G.L., Terblans J.J. (2012). Identification of Eu Oxidation States in a Doped Sr_5_(PO_4_)_3_F Phosphor by TOF-SIMS Imaging. Opt. Express.

[49] Tan X., Fan Q., Wang X., Grambow B. (2009). Eu(III) Sorption to TiO_2_ (Anatase and Rutile): Batch, XPS, and EXAFS Studies. Environ. Sci. Technol..

[50] Vercaemst R., Poelman D., Fiermans L., Van Meirhaeghe R.L., Laflère W.H., Cardon F. (1995). A Detailed XPS Study of the Rare Earth Compounds EuS and EuF3. J. Electron Spectrosc. Relat. Phenom..

[51] Nunn N., d’Amora M., Prabhakar N., Panich A.M., Froumin N., Torelli M.D., Vlasov I., Reineck P., Gibson B., Rosenholm J.M. (2018). Fluorescent Single-Digit Detonation Nanodiamond for Biomedical Applications. Methods Appl. Fluoresc..

[52] Shenderova O., Panich A.M., Moseenkov S., Hens S.C., Kuznetsov V., Vieth H.-M. (2011). Hydroxylated Detonation Nanodiamond: FTIR, XPS, and NMR Studies. J. Phys. Chem. C.

[53] Albers P.W., Klein H., Lox E.S., Seibold K., Prescher G., Parker S.F. (2000). INS-, SIMS- and XPS-Investigation of Diesel Engine Exhaust Particles. Phys. Chem. Chem. Phys..

[54] Gaur P., Banerjee S. (2019). C-N Cross Coupling: Novel Approach towards Effective Aryl Secondary Amines Modification on Nanodiamond Surface. Diam. Relat. Mater..

[55] Haasz A.A., Chiu S., Pierre J.E., Gudimenko Y.I. (1996). Thermo-Oxidative Erosion of Amorphous Hydrogenated Carbon Films. J. Vac. Sci. Technol. A Vac. Surf. Films.

[56] Sharin P.P., Sivtseva A.V., Popov V.I. (2021). Composition and Chemical State of Nanopowder Particles Obtained by Grinding Natural Diamond and by Detonation Synthesis. IOP Conf. Ser. Mater. Sci. Eng..

[57] Greczynski G., Hultman L. (2017). C 1s Peak of Adventitious Carbon Aligns to the Vacuum Level: Dire Consequences for Material’s Bonding Assignment by Photoelectron Spectroscopy. ChemPhysChem.

[58] Crunteanu A., Charbonnier M., Romand M., Vasiliu F., Pantelica D., Negoita F., Alexandrescu R. (2000). Synthesis and Characterization of Carbon Nitride Thin Films Obtained by Laser Induced Chemical Vapour Deposition. Surf. Coat. Technol..

[59] Egelhoff W.F. (1984). N_2_ on Ni(100): Angular Dependence of the N1s XPS Peaks. Surf. Sci..

[60] Marton D., Boyd K.J., Al-Bayati A.H., Todorov S.S., Rabalais J.W. (1994). Carbon Nitride Deposited Using Energetic Species: A Two-Phase System. Phys. Rev. Lett..

[61] Souto S., Alvarez F. (1997). The Role of Hydrogen in Nitrogen-Containing Diamondlike Films Studied by Photoelectron Spectroscopy. Appl. Phys. Lett..

[62] Sobaszek M., Brzhezinskaya M., Olejnik A., Mortet V., Alam M., Sawczak M., Ficek M., Gazda M., Weiss Z., Bogdanowicz R. (2023). Highly Occupied Surface States at Deuterium-Grown Boron-Doped Diamond Interfaces for Efficient Photoelectrochemistry. Small.

[63] Shames A.I., Panich A.M., Arnault J.-C. (2017). Paramagnetic Defects in Nanodiamonds. Nanodiamonds. Advanced Material Analysis, Properties and Applications.

[64] Panich A.M. (2012). Nuclear Magnetic Resonance Studies of Nanodiamonds. Crit. Rev. Solid State Mater. Sci..

[65] Shames A.I., Panich A.M., Kempiński W., Alexenskii A.E., Baidakova M.V., Dideikin A.T., Osipov V.Y., Siklitski V.I., Osawa E., Ozawa M. (2002). Defects and Impurities in Nanodiamonds: EPR, NMR and TEM Study. J. Phys. Chem. Solids.

[66] Shames A.I., Panich A.M., Porro S., Rovere M., Musso S., Tagliaferro A., Baidakova M.V., Osipov V.Y., Vul’ A.Y., Enoki T. (2007). Defects Localization and Nature in Bulk and Thin Film Utrananocrystalline Diamond. Diam. Relat. Mater..

[67] Abragam A. (1989). The Principles of Nuclear Magnetism.

[68] Goldman M. (1970). Spin Temperature and Nuclear Magnetic Resonance in Solids.

[69] Phillips J.C. (1996). Stretched Exponential Relaxation in Molecular and Electronic Glasses. Rep. Prog. Phys..

[70] Panich A.M., Shames A.I., Tsindlekht M.I., Osipov V.Y., Patel M., Savaram K., He H. (2016). Structure and Magnetic Properties of Pristine and Fe-Doped Micro- and Nanographenes. J. Phys. Chem. C.

[71] Dubois M., Guérin K., Petit E., Batisse N., Hamwi A., Komatsu N., Giraudet J., Pirotte P., Masin F. (2009). Solid-State NMR Study of Nanodiamonds Produced by the Detonation Technique. J. Phys. Chem. C.

[72] Shames A.I., Panich A.M., Osipov V.Y., Aleksenskiy A.E., Vul’ A.Y., Enoki T., Takai K. (2010). Structure and Magnetic Properties of Detonation Nanodiamond Chemically Modified by Copper. J. Appl. Phys..

[73] Shames A.I., Yu Osipov V., von Bardeleben H.J., Vul’ A.Y. (2012). Spin *S* = 1 Centers: A Universal Type of Paramagnetic Defects in Nanodiamonds of Dynamic Synthesis. J. Phys. Condens. Matter.

[74] Brodbeck C.M., Iton L.E. (1985). The EPR Spectra of Gd^3+^ and Eu^2+^ in Glassy Systems. J. Chem. Phys..

[75] Havlák L., Bárta J., Buryi M., Jarý V., Mihóková E., Laguta V., Boháček P., Nikl M. (2016). Eu^2+^ Stabilization in YAG Structure: Optical and Electron Paramagnetic Resonance Study. J. Phys. Chem. C.

[76] Zhang H., Lü M., Xiu Z., Wang S., Zhou G., Zhou Y., Wang S., Qiu Z., Zhang A. (2007). Synthesis and Photoluminescence Properties of a New Red Emitting Phosphor: Ca_3_(VO_4_)_2_:Eu^3+^;Mn^2+^. Mater. Res. Bull..

[77] Zou Z., Guo T., Gong G., Lin F., Sun Y., Huang J., Liao J., Wen H. (2023). Site Regulation and Luminescence Properties of Eu^3+^, Eu^2+^ in (La, Mg):SrAl_12_O_19_ Matrix. Spectrochim. Acta Part A Mol. Biomol. Spectrosc..

[78] Zhao M., Zhang Q., Xia Z. (2020). Structural Engineering of Eu^2+^ -Doped Silicates Phosphors for LED Applications. Acc. Mater. Res..

[79] Vul’ A.Y., Shenderova O.A. (2014). Detonation Nanodiamonds: Science and Applications.

[80] Yudina E.B., Romanov P.A., Chizhikova A.S., Aruev N.N. (2023). Pyrolysis mass-spectrometry study of detonation nanodiamonds surface chemistry. Fuller. Nanotub. Carbon Nanostruct..

[81] Brown I.D. (2005). The Chemical Bond in Inorganic Chemistry: The Bond Valence Model.

[82] Dang P., Liu D., Li G., Liang S., Lian H., Shang M., Lin J. (2019). Mixing the Valence Control of Eu^2+^/Eu^3+^ and Energy Transfer Construction of Eu^2+^/Mn^2+^ in the Solid Solution (1 − *x*)Ca_3_(PO_4_)_2_–*x*Ca_9_Y(PO_4_)_7_ for Multichannel Photoluminescence Tuning. Inorg. Chem. Front..

[83] Kumar S., Prakash R., Choudhary R.J., Phase D.M. (2015). Structural, XPS and Magnetic Studies of Pulsed Laser Deposited Fe Doped Eu_2_O_3_ Thin Film. Mater. Res. Bull..

[84] Li H., Wang Y. (2017). Effect of Oxygen Vacancies on the Reduction of Eu^3+^ in Mg_3_Ca_3_(PO_4_)_4_ in Air Atmosphere. Inorg. Chem..

