# Supplementary material for: Unexpected Reduction of Eu3+ Ions to Eu2+ on a Nanodiamond Surface: An XPS, NMR, EPR, and Photoluminescence Study
Source: Nanomaterials (Basel). 2026 Sep 1;16(17):1104. doi: 10.3390/nano16171104 (PMC13567252; doi:10.3390/nano16171104)
Supplement: Supplementary file 1 [file nanomaterials-16-01104-s001.zip › nanomaterials-4434269-supplementary.pdf]

Supplementary Materials
Unexpected Reduction of Eu^{3+} Ions to Eu^{2+} on the Nanodiamond Surface: XPS, NMR, EPR, and Photoluminescence study

N. Froumin[a], A.I. Shames[b], A. Chizhikova[c], M. Uchaev [c], A.M. Panich[b]*

[a] *Ilse Katz Institute for Nanoscale Science and Technology, Ben-Gurion University of the Negev, Beer-Sheva 84105, Israel*

[b] *Department of Physics, Ben-Gurion University of the Negev, 8410501 Beer-Sheva, Israel,*

[c] *Ioffe Institute, St. Petersburg 194021, Russia*

Infrared spectra

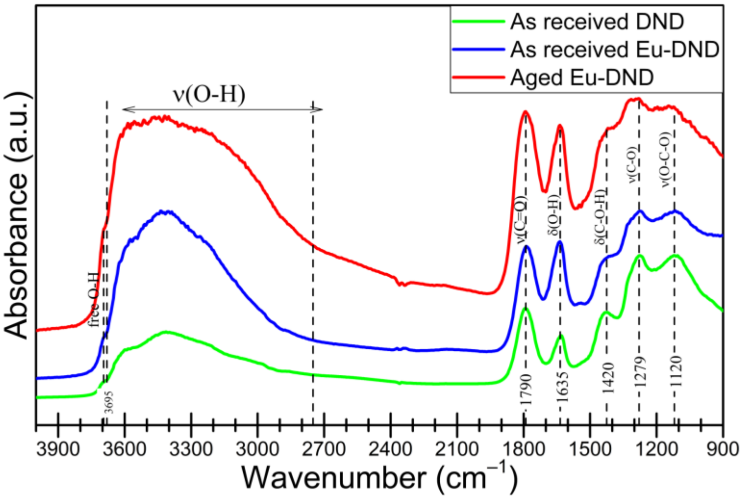

Figure S1. IR-spectra of DND and Eu-DND powders.

Figure S1 shows the infrared (IR) absorption spectra. The broad band observed in the range of 3695–2750 cm^{-1} is associated with the stretching vibrations of hydroxyl (OH) groups in adsorbed water molecules and is present in the IR spectra of all samples. Over time, the intensity of this band increases, reflecting an increase in the amount of adsorbed water molecules in Eu-DND samples. A narrower absorption band around 1635 cm^{-1} corresponds to the deformation vibrations of O–H bonds in water. Bands centered around 1115 cm^{-1} are attributed to O–C–O vibrations, originating from these groups on the DND surface. Strong absorption bands in the ranges of 1730–1796 cm^{-1} and 1268 cm^{-1} are characteristic of C=O stretching vibrations in the carboxyl group and C–O stretching vibrations in the same group, respectively [S1,S2]. The band at 1420 cm^{-1} corresponds to C–O–H bending vibrations in the carboxyl group.

References

S1. Detonation Nanodiamonds: Science and Applications; Vul', A.Y., Shenderova, O.A., Eds.; Pan Stanford Publishing: Singapore, 2014; ISBN 978-981-4411-27-1.
S2. Yudina, E.B.; Romanov, P.A.; Chizhikova, A.S.; Aruev, N.N. Pyrolysis Mass-Spectrometry Study of Detonation Nanodiamonds Surface Chemistry. Fullerenes, Nanotubes and Carbon Nanostructures 2023, 31, 68–74, doi:10.1080/1536383X.2022.2120477.
